# Effect of gargling with tea and ingredients of tea on the prevention of influenza infection: a meta-analysis

**DOI:** 10.1186/s12889-016-3083-0

**Published:** 2016-05-12

**Authors:** Kazuki Ide, Hiroshi Yamada, Yohei Kawasaki

**Affiliations:** Department of Drug Evaluation & Informatics, Graduate School of Pharmaceutical Sciences, University of Shizuoka, 52-1 Yada, Suruga-ku, Shizuoka 422-8526 Japan

**Keywords:** Tea, Catechins, Influenza, Infection, Hygiene, Meta-analysis

## Abstract

**Background:**

Influenza viruses can spread easily from person to person, and annual influenza epidemics are serious public health issues worldwide. Non-pharmaceutical public health interventions could potentially be effective for combatting influenza epidemics, but combined interventions and/or interventions with greater effectiveness are needed. Experimental studies have reported that tea and its ingredients (especially catechins) have antiviral activities. Although several clinical studies have investigated the use of tea or its ingredients to prevent influenza infections, the effect of gargling these substances has remained uncertain.

**Methods:**

We conducted a meta-analysis of randomized controlled studies and prospective cohort studies to assess the effect of gargling with tea and its ingredients on the prevention of influenza infection. The published literature was searched using the Cochrane Library, PubMed/MEDLINE (1966 to September 2015), Web of Science (1981 to September 2015), and Ichu-shi Web (1983 to September 2015). The extracted studies were read by two reviewers independently, and their overall scientific quality was evaluated. Studies meeting our inclusion criteria were pooled using the Mantel-Haenszel method in a fixed effects model and were also analyzed in a random effects model. The qualities of the model fits were assessed using the Akaike information criterion (AIC) and Bayesian information criterion (BIC).

**Results:**

The literature search and review identified 5 studies that met the inclusion criteria for the meta-analysis (total number of participants, 1890; mean age range, 16–83 years). The participants who gargled with tea or its ingredients showed a lower risk of influenza infection than did participants who gargled with placebo/water or who did not gargle (fixed effects model, Mantel-Haenszel method: relative risk [RR] = 0.70, 95 % confidence interval [CI] = 0.54–0.89; random effects model: RR = 0.71, 95 % CI = 0.56–0.91). The fixed effects model had a better quality of fit than the random effects model (fixed effects model: AIC = 6.04, BIC = 5.65; random effects model: AIC = 8.74, BIC = 7.52).

**Conclusions:**

Gargling with tea and its ingredients may have a preventative effect for influenza infection. However, additional large-scale studies in different populations and a pooled analysis of these studies are needed to confirm the effect.

## Background

Influenza viruses can spread easily from person to person, and annual epidemics create serious public health problems worldwide. The World Health Organization estimates that 5 to 10 % of adults and 20 to 30 % of children are infected by the virus annually, resulting in approximately 3 to 5 million cases of severe illness and 250,000 to 300,000 deaths [1]. Effective preventative measures are therefore needed to reduce serious cases of influenza.

Vaccination is a commonly used and widely recommended preventive measure for influenza infection [1, 2]. Its effectiveness has been reported in both randomized controlled trials and meta-analyses [2, 3]. Nonetheless, there are several drawbacks to vaccinations for influenza viruses, including the limited supplies of vaccines [4–6] and their strain-specific effects [2, 3]. The effectiveness of influenza vaccination may also depend on the characteristics of the vaccinated population [7]. Neuraminidase inhibitors are also used for influenza prevention, but they have limited effects and the existence of resistant viruses has been reported [8, 9]. Therefore, in addition to these pharmaceutical interventions, non-pharmaceutical public health interventions are also important for epidemic control, including measures such as the use of facemasks, hand hygiene, and gargling [10–12]. One meta-analysis study reported that a combination of hand hygiene with facemasks was effective for influenza prevention, even though hand hygiene was ineffective by itself [13]. Non-pharmaceutical public health interventions have the potential to be effective, but a combination of these interventions and/or improvements to their effectiveness would be needed to control influenza.

Tea catechins have been reported to have antiviral activities in *in vitro* and *in vivo* studies [14–16]. *In vitro* studies have suggested that (-)-epigallocatechin gallate, a highly bioactive catechin, reduces the infectivity of both influenza A and B viruses in Madin-Darby canine kidney cells [14, 15]. Influenza A and B are mainly spread seasonally, and are particularly problematic to public health. Several clinical studies have also investigated the use of tea and its ingredients to prevent influenza infection [17–23]. These studies have examined both the effects of tea consumption and the use of tea as a gargle rinse. However, only a limited number of studies have focused on tea consumption [20, 23], and the effect of gargling with tea or its ingredients has remained inconclusive. Indeed, although several small-scale studies have demonstrated efficacy [17, 18], other studies have not shown any significant effect [19, 21, 22].

Considering the current state of research, we decided to conduct a meta-analysis of randomized controlled studies and prospective cohort studies to assess the effect of gargling with tea and its ingredients on the prevention of influenza infection.

## Methods

### Design overview

The primary endpoint of this study was influenza infection that had been confirmed by antigen detection testing and/or antibody titer testing. We searched the published literature using common electronic databases (Cochrane Library, PubMed/MEDLINE, Web of Science, and Ichu-shi Web), and additionally performed a manual search using the reference lists of the included studies. All studies were reviewed by two reviewers independently (KI and HY). The overall scientific quality of the randomized controlled studies was assessed using the Jadad score [24, 25] and the Cochrane Collaboration's tool for assessing risk for bias [26]. The non-randomized cohort studies were assessed using the Newcastle-Ottawa scale [27, 28]. The studies that were ultimately selected for our meta-analysis were pooled using the Mantel-Haenszel method [29] and a random effects model. Publication bias was assessed using a funnel plot [30] and Egger’s regression analysis [31]. This study was a meta-analysis of previously published results and did not enroll any humans or animals. Accordingly, additional informed consent and ethical approval were not required.

### Search strategy and selection criteria

The published literature was searched using the Cochrane Library (http://www.cochranelibrary.com/), PubMed/MEDLINE (1966 to September 2015), Web of Science (1981 to September 2015), and Ichu-shi Web (1983 to September 2015). This search was performed with the following terms in MEDLINE: (“catechin” [MeSH Terms] OR “tea” [MeSH Terms]) AND (“influenza, human” [MeSH Terms] OR “upper respiratory tract inflammation” [All Fields]) AND gargling [All Fields] AND (clinical trial [pt] OR “clinical trials as topic” [MeSH Terms: noexp] OR trial [ti]) AND Clinical Trial [ptyp]. In addition, the reference lists of the included studies were searched manually.

All studies extracted from the databases were independently reviewed by two reviewers (KI and HY). As noted above, the randomized controlled studies were assessed using the Jadad score [24, 25] and the Cochrane Collaboration's tool for assessing risk for bias [26]. The Jadad score is calculated by assessing randomization (range, 0–2), double blinding (range, 0–2), and withdrawals and dropouts (range, 0–1). The total score ranges from 0 to 5 and is interpreted according to the following criteria: 0–2 indicates a low-quality report and 3–5 indicates a high-quality report. The non-randomized cohort studies were assessed using the Newcastle-Ottawa scale [27, 28]. This scale is constructed from 3 grouping items, and stars are awarded for each item: 1) selection of cohorts (range, 0–4 stars), 2) comparability of cohorts (range, 0–2 stars), and 3) assessment of outcomes (range, 0–3 stars). The total number of stars ranges from 0 to 9, and a high quality study can be awarded 9 stars.

The meta-analysis included studies that met each of the following criteria: 1) studies with randomized controlled designs or prospective cohort designs; 2) studies that investigated gargling with tea or its components for at least 60 days; 3) studies with a control group, such as a placebo, water, or non-gargling group; 4) studies in which influenza infection was confirmed by antigen and/or antibody detection methods; 5) studies published in English and/or Japanese. The criterion regarding the duration of the gargling was chosen based on seasonal epidemic periods.

### Data extraction

The following information was extracted from published reports: 1) the authors’ names and the year of publication, 2) the study population, 3) the study design and the duration of the intervention or observation, and 4) the number of participants infected by influenza and the methods of confirmation.

### Endpoint

The primary endpoint of this study was influenza infection that had been confirmed by an antigen detection test. In addition to rapid antigen tests that are used in clinical practice, we also included changes in antibody titer in this endpoint.

### Statistical analyses

The studies that were selected for the meta-analysis were pooled using both the Mantel-Haenszel method for a fixed effects model and a random effects model [32, 33]. The random effects model was fitted via restricted maximum-likelihood estimation. The results were expressed as relative risks (RRs) with 95 % confidence intervals (CIs). Heterogeneity among the studies was assessed using a *Q*-test and quantified with the *I*^2^ statistic for the random effects model [34]. For the *Q*-test, *P* < 0.1 was considered representative of statistically significant heterogeneity. *I*^2^ estimates the total percentage of variability in the effect size estimates, with *I*^2^ > 50 % indicating moderate to large heterogeneity. The relative qualities of model fits were assessed using the Akaike information criterion (AIC) [35] and the Bayesian information criterion (BIC) [36]. AIC and BIC values were compared between the fixed and random effects models to judge their relative qualities, with smaller values of AIC and BIC indicating better fits of the model to the data [35, 36]. Publication bias was assessed using a funnel plot [30] and Egger’s regression analysis [31]. For the Egger’s regression analysis, *P* < 0.1 was considered representative of statistically significant publication bias. All statistical analyses were performed using R version 3.2.0 for Windows with the Metafor package [37] (The R Foundation for Statistical Computing, Vienna, Austria).

## Results

### Description of the included studies

**Fig. 1:**
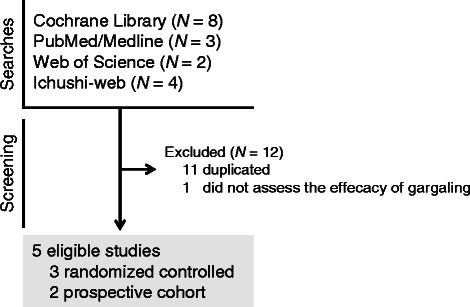
Flow diagram for the study

The flow diagram for the meta-analysis is shown in Fig. 1. Seventeen studies were identified in the literature search (8 from the Cochrane Library, 3 from PubMed/Medline, 2 from Web of Science, and 4 from Ichu-shi Web). Twelve studies were excluded because they were duplicated or did not present original data on the efficacy of gargling. The 5 remaining studies were included in the meta-analysis. Of the included studies, 2 had prospective cohort designs and 3 were randomized controlled trials. The characteristics of the studies, such as the publishing details (author and year), study populations, and study designs, are shown in Table 1. The total number of participants in each study ranged from 124 to 757, and the range of mean ages was 16 to 83 years. The durations of the studies ranged from 3 to 5 months. In the randomized controlled studies, the active groups received either green tea extract (catechin solution, 1 study) or bottled green tea (2 studies), and the control groups received either placebo (1 study) or water (2 studies). One cohort study selected the exposed cohort from the population of persons with a habit of gargling green tea. The other cohort study selected the exposed cohort from the population of persons with a habit of gargling black tea. The 2 cohort studies selected the non-exposed cohort from the same population—persons with or without a habit of gargling water. All of the 5 studies were conducted in Japan.

**Table 1:** Characteristics of the included studies

			Age (yr), mean ± SD		Sex (M/F)		Duration (mo)	Study design	Reference
Author	Year of publication	Total *N*	Tea	Control	Tea	Control			
Iwata *et al.*	1997	297	30 ± NA	24 ± NA	NA	NA	5	Prospective cohort	[17]
Yamada *et al.*	2006	124	83 ± 8	83 ± 8	24/52	16/32	3	Prospective cohort	[18]
Yamada *et al.*	2007	404	40 ± 11	40 ± 12	36/164	52/152	3	Randomized controlled	[19]
Toyoizumi *et al.*	2013	308	16 ± 1	16 ± 1	98/57	86/67	3	Randomized controlled	[21]
Ide *et al.*	2014	757	16 ± 1*	16 ± 1*	224/160*	199/164*	3	Randomized controlled	[22]

*yr* years, *SD* standard deviation, *M* male, *F* female, *mo* months, *NA* not assessed.

* Full analysis set (Characteristics of the total population is not shown in the article)

Regarding the overall scientific quality of the studies, the 2 prospective cohort studies were awarded 7 stars on the Newcastle-Ottawa scale, and the 3 randomized controlled studies received Jadad scores of 3 to 5. The results of the assessments using the Newcastle-Ottawa scale are shown in Table 2 and, the results of those using the Cochrane Collaboration's tool for assessing risk for bias and the Jadad score are shown in Table 3.

**Table 2:** Overall scientific quality of the prospective cohort studies

*Newcastle-Ottawa scale*		
Study	Iwata *et al.* 1997	Yamada *et al.* 2006
Criterion	[17]	[18]
Selection (range, 0–4 stars)	***	***
Comparability (range, 0–2 stars)	*	*
Outcome (range, 0–3 stars)	***	***

* One asterisk represents one star

**Table 3:** Overall scientific quality of the randomized controlled studies

Study	Yamada *et al.* 2007	Toyoizumi *et al.* 2013	Ide *et al.* 2014
Criterion	[19]	[21]	[22]
*Cochrane Collaboration's tool for assessing risk for bias*			
Random sequence generation	Low	Low	Low
Allocation concealment	Low	High	High
Blind participants and personnel	Low	High	High
Blind outcome assessment	Low	Low	Low
Incomplete outcome data	Low	Low	Low
Selective reporting	Low	High	High
Other bias	Low	Unclear	Unclear
*Jadad score*			
Randomization (range, 0–2)	2	2	2
Double blinding (range, 0–2)	2	0	0
Withdrawals and dropouts (range, 0–1)	1	1	1
Total score	5	3	3

### Heterogeneity

The *Q*-test and *I*^2^ statistic both suggested low heterogeneity among the studies included in this meta-analysis (*I*^2^ = 0.00 %; *Q*-test: *Q* = 3.05, *P* = 0.55).

### Efficacy assessment

**Fig. 2:**
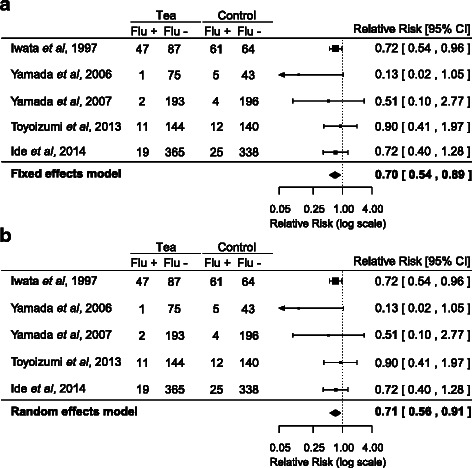
Forest plot of fixed effects model (**a**) and random effects model (**b**)

A forest plot of the RRs for influenza infection is shown in Fig. 2a, b. The 5 studies included a total of 1890 participants. The participants who gargled with tea or its ingredients showed a lower risk of influenza infection than did the participants who gargled with placebo/water or who did not gargle (fixed effects model: RR = 0.70, 95 % CI = 0.54–0.89; random effects model: RR = 0.71, 95 % CI = 0.56–0.91). The results were consistent between the fixed effects model (Fig. 2a) and the random effects model (Fig. 2b). The qualities of the model fits were as follows: the fixed effects model had an AIC of 6.04 and a BIC of 5.65, while the random effects model had an AIC of 8.74 and a BIC of 7.52. Accordingly, the fixed effects model showed a better quality of fit to the data, as compared with the random effects model.

### Potential publication bias

**Fig. 3:**
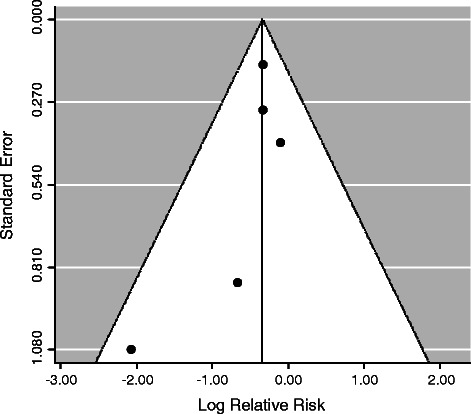
Funnel plot

A funnel plot of the included studies is shown in Fig. 3. Because of the limited number of studies that were included in this analysis (*n* = 5), visual interpretation of the plot was inconclusive. The result of the Egger’s regression analysis was not significant (*P* = 0.30).

## Discussion

The meta-analysis of 5 studies with 1890 participants indicated that gargling with tea and its ingredients reduces the risk of influenza infection, as compared with the control activities. The effects of gargling with tea and its ingredients on the prevention of influenza infection have been investigated since the 1990s; nonetheless, the present study is the first meta-analysis to consider this subject. A combination of non-pharmaceutical interventions and/or an improvement in the effectiveness of non-pharmaceutical interventions are needed to control influenza epidemics [13]. Tea and its ingredients may be useful for this purpose. The mean ages of participants in the included studies ranged from 16 to 83 years, and this suggested that gargling with tea and its ingredients might be effective for any age group or population. All of the studies included in the analysis used a control group or non-exposed cohort from the same population, which ensures the comparability of their results. In addition, all of the cohort studies and randomized controlled trials were well designed [17–19, 21, 22]. Therefore, the results of this meta-analysis of well-designed studies may be reliable. The AIC and BIC values for the fixed effects model were smaller than those for the random effects model, which indicates that the fixed effects model had a better fit.

Although the meta-analytic results of this study suggest that gargling with tea and its ingredients has efficacy for influenza prevention, there are several limitations to our study. A principal limitation is the small number of studies that met the inclusion criteria. Further, all 5 of these studies were conducted in Japan, which could bias or limit interpretations of the findings. The fact that all of the studies were conducted in Japan may have helped to avoid between-study heterogeneity, which is often caused by the inclusion of different populations with different sociodemographic characteristics. Nonetheless, additional studies of different populations would still be needed to improve the generalizability of the meta-analytic results. If the number of studies were increased, a sensitivity analysis could also be performed. Sensitivity analyses can provide indications of the robustness of meta-analytic results, including insight into factors that affect the efficacy of gargling with tea and its ingredients [38, 39]. A sensitivity analysis should be carried out in future pooled analyses with additional high-quality studies. As additional high-quality studies become available in the future, it may also be possible to consider more sophisticated methods for the meta-analysis. We used standard methods for the meta-analysis in the present study [32, 33]. However, several newer approaches are also available, such as individual participant data meta-analysis [Riley 2010] and Bayesian meta-analysis [Sutton 2008]. If future meta-analytic studies apply both standard and new approaches, then they could offer more reliable results and greater information about the robustness of the meta-analytic conclusions. The quality of the present study could also be improved by expanding the analysis to additional databases, and by collecting data from other Asian countries. Although we searched 5 major databases for the present study, there are several databases that were not searched, such as EMBASE.

With respect to publication bias, the result of the Egger’s regression analysis was not significant and therefore did not suggest significant publication bias. Nonetheless, visual interpretation of the funnel plot was inconclusive. The results may have been affected by the small number of studies that were pooled in the analysis, and additional studies are needed to obtain more definitive conclusions regarding publication bias. In the future, additional large-scale randomized controlled studies and a pooled analysis of these studies could help to improve the accuracy and robustness of the effect estimate for gargling with tea and its ingredients.

## Conclusions

Gargling with tea or ingredients of tea may be effective for the prevention of influenza infection. Given the effectiveness that has been shown in this meta-analysis, gargling with tea or its ingredients may provide a simple and useful addition to the non-pharmaceutical interventions that are currently employed for influenza control. However, additional large-scale randomized controlled trials of different populations are needed to confirm our findings.

### Ethics and consent to participate

This study was a meta-analysis of previously published results and did not enroll any humans or animals. Accordingly, additional informed consent and ethical approval were not required.

### Consent for publication

Not Applicable.

### Availability of data and materials

No additional data available.

## Abbreviations

AIC: Akaike information criterion
BIC: Bayesian information criterion
CI: confidence interval
RR: relative risk
